# Disrespect and abuse of women during childbirth in Nigeria: A systematic review

**DOI:** 10.1371/journal.pone.0174084

**Published:** 2017-03-21

**Authors:** Foluso Ishola, Onikepe Owolabi, Veronique Filippi

**Affiliations:** 1 Atlas Service Corps, Washington, District of Columbia, United States of America; 2 International Centre for Evaluation and Development, Nairobi, Kenya; 3 Faculty of Epidemiology and Population Health, London School of Hygiene and Tropical Medicine, London, United Kingdom; 4 Guttmacher Institute, New York, United States of America; University College London, UNITED KINGDOM

## Abstract

**Background:**

Promoting respectful care at childbirth is important to improve quality of care and encourage women to utilize skilled delivery services. However, there has been a relative lack of public health research on this topic in Nigeria. A systematic review was conducted to synthesize current evidence on disrespect and abuse of women during childbirth in Nigeria in order to understand its nature and extent, contributing factors and consequences, and propose solutions.

**Methods:**

Five electronic databases were searched for relevant published studies, and five data sources for additional grey literature. A qualitative synthesis was conducted using the Bowser and Hill landscape analytical framework on disrespect and abuse of women during childbirth.

**Results:**

Fourteen studies were included in this review. Of these studies, eleven were cross sectional studies, one was a qualitative study and two used a mixed method approach. The type of abuse most frequently reported was non-dignified care in form of negative, poor and unfriendly provider attitude and the least frequent were physical abuse and detention in facilities. These behaviors were influenced by low socioeconomic status, lack of education and empowerment of women, poor provider training and supervision, weak health systems, lack of accountability and legal redress mechanisms. Overall, disrespectful and abusive behavior undermined the utilization of health facilities for delivery and created psychological distance between women and health providers.

**Conclusion:**

This systematic review documented a broad range of disrespectful and abusive behavior experienced by women during childbirth in Nigeria, their contributing factors and consequences. The nature of the factors influencing disrespectful and abusive behavior suggests that educating women on their rights, strengthening health systems to respond to specific needs of women at childbirth, improving providers training to encompass interpersonal aspects of care, and implementing and enforcing policies on respectful maternity care are important. This review has also shown that more robust research is needed to explore disrespect and abuse of women during childbirth in Nigeria and propose compelling interventions.

## 1. Introduction

Out of an estimated 303,000 maternal deaths that occurred worldwide in 2015, 99% occurred in low-and middle income countries [1, 2]. Nigeria with its large population and high fertility rate contributed 19% of global maternal deaths, with an estimated 58,000 deaths occurring in 2015. It also has a high maternal mortality ratio (MMR) estimated at 814 maternal deaths per 100,000 live births and lags behind many countries in the region[3]. A factor contributing to this high MMR is the low rate of skilled birth attendance with 45% of births in Nigeria attended by skilled health personnel in 2015[3].

Ensuring skilled birth attendance for all deliveries is a key strategy to reducing global, regional and national maternal mortality ratio [4], and target three of the Sustainable Development Goals (SDG) [5]. Achieving this requires overcoming financial, cultural, economic and geographic barriers to skilled birth attendants at delivery, as well as improving the quality of care received by women [6, 7]. While access to routine maternity care is not yet guaranteed for many women during childbirth in Nigeria, recent studies indicate that women using skilled birth attendants at delivery are subjected to poor quality of care in form of abusive and disrespectful care [8, 9].

Respectful care during childbirth has been described as “a universal human right that encompasses the principles of ethics and respect for women’s feelings, dignity, choices and preferences”[10–12]. Disrespect and abuse during childbirth infringes on these basic principles of human rights and violates the fundamental obligation to provide support and healing [13–15]. Different attempts have been made to define and categorize this topic. Bowser and Hill (2010), in a landscape analysis, categorized disrespectful and abusive care at childbirth into seven types: physical abuse, non-consented care, non-confidential care, non-dignified care, discrimination, abandonment of care and detention in facilities[16]. Freedman et al. (2014) [17] asserted that this topic has not been clearly defined and proposed a model to incorporate individual, structural and policy level factors. They defined disrespect and abuse during childbirth as “interactions or facility conditions that local consensus deems to be humiliating or undignified, and those interactions or conditions that are experienced as or intended to be humiliating or undignified” [17]. Bohren et al. (2015) [18], in their systematic review, further developed an evidence-based typology of the mistreatment of women during childbirth in health facilities in an effort to inform the development and application of measurement tools for assessing this phenomenon. This was categorized into seven domains: physical abuse, sexual abuse, verbal abuse, stigma and discrimination, failure to meet professional standard of care, poor rapport between women and provides, and health system conditions and restraints.

While the terminology “disrespect and abuse” or “mistreatment” related to childbirth was recently introduced and conceptualized in 2010 and 2015 respectively [16, 18], research related to the subject has been going on for many years [19–29]. These studies support assertions that disrespect and abuse of women during childbirth has been in existence for a while yet it has received little programmatic attention until recently [14].

This important negative aspect of maternity care can influence women’s decision not to make use of health facilities in their present or subsequent deliveries [7, 21, 30] thus contributing to the number of births assisted by non-skilled personnel. It is therefore crucial to know what forms of disrespect and abuse exist and how to prevent them, and better meet women’s emotional, physical, socio-cultural and psychological needs as part of broader efforts to provide better quality care.

In addition, disrespect and abuse of women at childbirth in the Nigerian health system has not been comprehensively documented despite its possible importance to the reduction of Nigeria’s MMR [8, 19]. This review fills a gap in literature by synthesizing current quantitative and qualitative evidence to explore its forms, causes, consequences and proffer solutions. Detailed knowledge may help improve the quality of maternity care and increase the utilization of facilities for delivery in Nigeria.

## 2. Methods

A systematic review of published quantitative and qualitative literature between January 2004 and July 2014 was initially conducted to capture studies published in the last ten years. However, this period was extended till October 2015 to update our literature search before the final analysis and writing up was done. The Bowser and Hill classification informed the synthesis of this review as it provided a framework for the classification, contributing factors and consequences of disrespect and abuse during childbirth that fit the aim of this review.

Our analyses was conducted using Bowser and Hill’s framework to: 1) describe and understand the nature of disrespect and abuse of women during childbirth; 2) identify and analyze the factors contributing to and consequences of disrespect and abuse of women during childbirth; 3) propose recommendations on how to address disrespect and abuse of women during childbirth.

### 2.1 Search strategy

Potentially relevant articles for the systematic review were identified by searching bibliographical databases (Embase, Medline, Popline, Cinahl, and Africa Wide Information), the WHO Global health Library, organizational databases (White Ribbon Alliance, USAID, Maternal and Child Integrated Program (MCHIP), Health Policy Project, Population Council) and Google Scholar. A full search strategy for each database was developed using key words/ free text terms in various combinations for three concepts: (a) quality of care, disrespect or abuse, providers attitude; (b) intrapartum care, childbirth, labor; and (c)Nigeria. Medical Subject Headings (MeSH) or equivalent indexing terms were used to capture all relevant terms used by authors. Thesaurus or MeSH terms were applied to each synonym where applicable and exploded where appropriate (S1 Appendix). All searches were initially conducted between 16^th^ and 20^th^ of July 2014 and updated on October 31^st^ 2015 to allow the authors include new publications.

Additional publications were identified by hand searching bibliographies and related references from identified studies, and contacting researchers in this field for assistance to identify relevant studies.

### 2.2. Inclusion and exclusion criteria

FI screened titles, and abstracts of identified citations for potential inclusion in the review and full texts were sought for relevant articles. Studies were eligible for inclusion if they collected primary data, were conducted in Nigeria, reported on indicators that can be classified under disrespect and abuse of women during childbirth, reported on contributing factors or consequences of disrespect and abuse of women during childbirth, investigated quality of care directly or indirectly related to the disrespect and abuse of women during childbirth, aimed to understand and explore actual experiences of women during childbirth and reported any form of disrespect and abuse, reported reasons for non-utilization or delayed utilization of skilled delivery services involving any form of disrespect and abuse. Systematic reviews investigating interventions to address disrespect and abuse, studies published prior to 2004 and non-English studies were excluded.

### 2.3. Quality appraisal

FI appraised the included studies for relevance, reliability and rigor. The Centre for Evidence-Based Management (CEBM) Tool for Critical Appraisal of a Survey [31] was used for the cross sectional studies and the Critical Appraisal Skills Programme (CASP) Tool [32] was used to appraise the qualitative studies. The overall quality assessment was judged high, medium or low. No studies were removed as a result of the quality appraisal.

### 2.4. Data extraction

Data were extracted for each paper using standardized forms with the following domains; name of first author and year of publication, study location and setting, study design, study description, sample size and demographics, type and characteristics of disrespect and abuse, analysis method, main findings and limitations.

### 2.5. Data synthesis

Bowser and Hill framework was employed in the synthesis. The framework categorizes disrespect and abuse into seven domains earlier mentioned and categorizes contributing factors into; individual and community, policy, governance, providers and service delivery factors with consequent underutilization of skilled delivery services. The results were collated and analyzed under this categories to satisfy the key objectives of the review. For quantitative synthesis, we reported the type of disrespect and abuse experienced under each category in form of percentages. For qualitative synthesis, we presented participants’ quotations and author’s analysis that fit the theme of disrespect and abuse being reported.

## 3. Results

**Fig 1:**
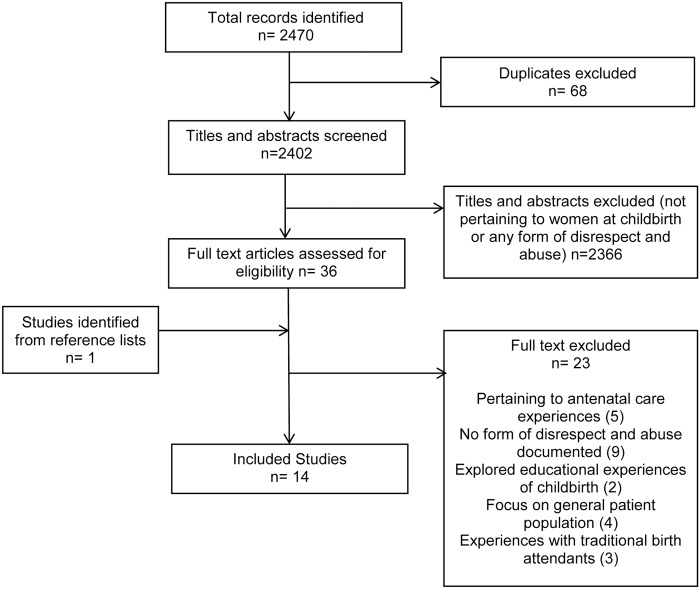
Flow chart of search and study inclusion process.

The initial search yielded 2115 citations and the updated search yielded an additional 355 making a total of 2470, of which 68 duplicates were removed, After title and abstract screening, 36 potentially relevant articles were identified for full text review. One additional eligible article was identified through manual searches of reference lists. This left a total of 37 studies to be screened at full text level. 14 studies met the inclusion criteria and 23 studies were excluded (Fig 1).

Among the 14 studies included, 11 were cross-sectional studies, 1 qualitative study and 2 mixed method studies. Ten studies were conducted in Southern [8, 33–41], and four in Northern Nigeria [42–45]. Five were done in rural settings [33–35, 42, 43] and nine in urban settings [8, 36–41, 44, 45]. Table 1 presents the summary results of the included studies.

**Table 1:** Summary results of included studies.

Author, year	Study location and setting	Study design and description	Sample size	Demographics	Type and characteristics of disrespect and abuse	Results	Type of analysis done	Bias/ Limitations
Igboanugo GM et al. 2011[33]	Niger Delta Rural	Qualitative study (Semi structured interviews)	8 pregnant women Purposive sampling of women who had previously delivered in a maternity center	Women age range 24–35 years	Bullying, Insult, lack of privacy, overcrowded labor rooms, delay in getting treatment	Negative attitude of staff towards pregnant women cited as main reason for avoiding maternity care	Analysis by grounded theory	Small sample size. Although internally valid, might be difficult to extrapolate results to other settings, so low external validity
Uzochukwu et al. 2004 [34]	Enugu State. Oji river LG May 1999	Mixed (Cross sectional study and Focused Group Discussion) Interviewer administered questionnaire	Survey of 405 households selected by simple random sampling. Four FGDs (9–10 members per group) by purposive sampling of women of who had delivered in the preceding 12 months	One woman per household who has had a delivery in the preceding twelve months. Households excluded if this criteria not met.	Bad and unfriendly attitude, insults without provocation, insensitive to patients, non-explanation of diagnosis or results	29.3% reported poor staff attitude. P< 0.05	Survey data analyzed by tabulations and FGDs transcribed and content analysis used	Unclear on precision without Confidence Intervals (CI’s). Open to selection bias as some households excluded. Also open to recall bias
Udoma E.J et al. 2008 [35]	Akwa Ibom and Cross River State. Feb to July 2003	Cross-sectional Study(Questionnaires)Interviewer completed questionnaire	2063 pregnant women from 47 spiritual church based clinics.	Women aged between 15 and 48 years	Unfriendly staff attitude	12.1% had experienced harsh attitude of health workers	Full details of analysis done not provided	No information on power calculations for sample size and sampling technique used. Statistical significance and CI not calculated
Moronkola O A et al. 2007[42]	Kogi State. Rural community	Cross-sectional Study (Questionnaires)	1,640 women from 11 rural local governments out of 21 LG available.	Women aged 15 to 49 years who had experienced childbirth	Poor attitude of health workers	64.3% reported poor attitude of health workers.X^2^ = 134.69, df(1) p<0.0001	Univariate analysis and chi2 tests for association	Unclear on precision without CI's. No information on power calculations for sample size and sampling technique used.
Moore BM et al. 2011 [36]	Rivers State. Urban facility	Cross-sectional study (Questionnaires)	112 mothers. 2 health centers selected from 12 health centers by systematic random sampling, 112 mothers were then selected by simple random sampling	Women aged 15 to 49 years	Unfriendly attitude of staff, rude, arrogant, neglectful behavior	70.8% reported unfriendly attitude of staff	Simple descriptive statistics in form of percentages	Inconsistency in the abstract and the study, methods not detailed. Results not generalizable to the population. Small sample size.
Iyaniwura et al. 2009 [39]	Sagamu, Ogun State. September to October 2005. Suburban	Cross sectional study (Structured questionnaire)	392 women selected by multistage sampling. 3 wards randomly selected from 11 and then five streets selected randomly from each ward. The women were then systematically selected from each street	Women of childbearing age who had carried at least one pregnancy to term in the past 5 years	Bad attitude of staff, long waiting time	11.3% reported bad attitude of staff. 29.4% cited long waiting times	Chi-square statistics to evaluate association between some variables. Results presented as percentages	Open to recall bias. Statistical significance and CIs not given for all variables
Lamina MA et al. 2004 [40]	Sagamu, Ogun State. Dec 2001- May 2002. Suburban	Cross sectional study (Structured questionnaires, self-administered)	266 women from the maternity clinic of the teaching hospital	Women aged 16 to 42 years with at least 1 delivery	Time wasting, bad attitude, lack of privacy	16.5% reported time wasting and bad attitude of hospital staff	Not detailed	Open to selection bias, No information on power calculation for sample size and sampling technique used. Statistical significance and CI not calculated, confounders not fully accounted for
Onah HE et al. 2006 [37]	Enugu State. April to June 2004. Urban	Cross sectional study (Structured questionnaires)	1098 women selected by multistage sampling. 3 districts selected randomly from 12 districts in the city, list of the houses in the 3 districts then obtained and 800 houses selected randomly	Women who had delivered in the last 3 months	Unfriendly behavior and rudeness of staff, promptness of care, lack of privacy	24.3% said attitude of staff p<0.001, 94.2% cited promptness of care p<0.001, and 6.8% cited lack of privacy p<0.001	Chi-square used as test of significance at 95% confidence level	CI not calculated, confounders not fully accounted for
Sule et al. 2012 [44]	Zaria Primary health center(PHC) Urban	Cross sectional Study (Questionnaires)	315 women, Sampling technique not given	Women attending the maternity clinic of a primary health facility who had had at least one delivery	Companionship not allowed during delivery, unfriendly attitude, not allowed to deliver in preferred position	12.05% was not allowed companionship, 10.84% cited unfriendly health workers, 4.82% prevented from being delivered in preferred position, 2% cited lack of privacy	Univariate analysis and chi2 tests for association	Open to selection bias, small sample size
Chigbu et al. 2011 [38]	Enugu State 3 maternity care centers Urban	Cross sectional study. Interviewer administered questionnaires	1008 women, Sampling technique not given	Pregnant women with at least one delivery	Denial of pain relief during labor	66.5% of women were denied pain relief during labor despite requesting for it	Fischer exact test at a 95% confidence level	Courtesy bias, most of the women were questioned by their providers
Idris et al. 2013 [45]	Kaduna State Semi-urban community	Cross sectional study. Structured interviewer administered questionnaire	150 mothers, Multi stage sampling technique	women of reproductive age group (15–49 years) who delivered in the 24 months preceding the survey	negative provider attitude	negative provider attitude reported by 23.7% (CI = 16.4–32.7) p< 0.0001	Frequencies and percentages with 95% confidence intervals and test of significance	Courtesy bias, recall bias, Confounders not fully accounted for
Okafor et al. 2015 [8]	Enugu State Teaching hospital	Cross sectional study. Self-administered, structured and pre tested questionnaire	446 women. Convenience sampling	Mothers accessing immunization for their new-born who had delivered in the previous 6 weeks.	Physical abuse Non consented care Non dignified care Detention in facilities Abandonment and neglect Discrimination Non confidential care	(98.0%) reported at least one kind of abuse and disrespect during their last childbirth	Cross tabulation to analyze categorical data, and relationships expressed using odds ratios and confidence intervals. p<0.05	Recall bias, small sample size, Results not generalizable to the population
Nnebue et al. 2014 [41]	Enugu State(Nnewi North LG) PHC facilities	Mixed (Cross sectional study and Focused Group Discussion) Interviewer administered pretested semi-structured questionnaire	280 women by multi stage sampling Four FGDs by purposive sampling	Women utilizing maternal health care services	Time wasting but good attitude of health care providers	45.7% reported long waiting time before being attended to during delivery	Frequency distributions presented in tables. Statistical significance using Chi-square test, P < 0.05.	Small sample size, recall bias. Courtesy bias, most of the women were questioned by their providers
Ashimi et al. 2015 [43]	Jigawa State Two general hospitals	Descriptive cross sectional study, pretested interviewer administered semi structured questionnaire	410 women by systematic sampling	Pregnant women attending antenatal clinic whose last birth was 15 months or less.	Poor attitude of health workers	12.9% reporting poor attitude of health workers as reason for home birth	Frequencies and percentages. Means and standard deviation. The chi-square test or Fishers test. P <0.05	Courtesy bias. Facility based, not generalizable to the community.

Abbreviations: Primary Health Centre (PHC), Focus Group Discussion (FGD), Local Government (LG), Confidence Interval (CI)

The frequency of disrespect and abuse was high in studies that reported on prevalence, ranging from 11% to 71%.

Using the Bowser and Hill framework we report our analysis under the following subtitles.

### 3.1. Physical abuse

In the study by Okafor et al. [8] women reported physical abuse during childbirth (36%). These included being “restrained or tied down during labor” (17.3%), “episiotomy given or sutured without anesthesia” (9.2%), being “beaten, slapped, or pinched” (7.2%); and being “sexually abused by health worker” (2%).

### 3.2. Non-consented care

In a mixed method study by Uzochukwu et al. in Enugu State [34], most women reported absence of patient information processes by their health care providers: “*We want nurses who are kind*, *who would be patient enough to tell us what is happening to us*. *Most of them (nurses) do not…*.” In another study, more than half of women (54.5%) also reported non-consented procedures such as labor augmentation, shaving of pubic hair, sterilization, caesarean delivery and blood transfusion[8].

### 3.3. Non-confidential care

Women reported non-confidential care in four studies including lack of privacy [8, 33, 37, 40], and disclosure of sensitive patient information without consent[8]. In the qualitative study by Igboanugo et al. [33] comprising 8 pregnant women purposively selected from the Niger Delta population, one of the women stated,” *There was not enough space in the labor room…and they don’t have the facilities*. *There were only two rooms and about 4 doctors…you could hear them (doctors) talking to other women*”. Lamina et al. in a cross sectional study in Ogun State reported 16.5% of women complaining about too many medical students around during delivery without any privacy [40]. Women also reported disclosure to third parties of age (16.1%), medical history (1.8%) and HIV status (1.8%) without consent [8].

### 3.4. Non-dignified care

Negative, poor and unfriendly provider attitude at their last delivery was reported by a range of 11.3% to 70.8% of women in eight cross-sectional surveys [8, 35–37, 39, 42, 43, 45]. Women reported insults without provocation and insensitiveness to patients [34]. According to one of the women in the mixed method study conducted in Enugu State “*I think the attitudes of our nurses are bad because they have no respect or mercy for a patient and they insult patients without been provoked*” [34]. Igboanugo et al. also reported women being bullied and insulted. The women were quoted as follows “*They (health workers) feel they are doing you a favor by coming to the hospital ………*.*feel like the hospital is a home for demons*, *because of the character of the workers*” [33]. However, in one study by Nnebue et al. [41], women reported good attitude of health workers, “*They are friendly and receptive; they listen to you and treat you well…*.”

### 3.5. Discrimination

In a cross-sectional study by Okafor et al. in Enugu State, 20% of women reported discrimination on the basis of ethnicity, low social class, young age and HIV seropositive status[8], however, in four cross sectional studies [35, 37, 39, 42], women of low socioeconomic status and with no formal education reported experiences of unfriendly and harsh attitudes of staff in higher proportion.

### 3.6. Abandonment/Neglect

In one cross-sectional study, 29.1% of women reported abandonment and neglect [8]. In another cross-sectional study by Sule et al., 12.1% of women were denied companionship during labor [44] whilst in the cross-sectional study by Chigbu et al., 66.5% of women were denied pain relief during labor despite requesting for it [38]. Lack of promptness of care and time wasting was reported by a range of 10% to 24% of women in three studies [37, 39, 40].

### 3.7. Detention in facilities

22% of women reported detention in facilities for failure to pay their bills and that of their babies only in the cross-sectional study by Okafor et al conducted in a teaching hospital Enugu State[8].

### 3.8. Factors contributing to disrespect and abuse during childbirth

Most of the included papers identified a range of factors contributing to disrespect and abuse in Nigeria according to the analysis they made of women’s perceptions or self-reports including:

### 3.9. Individual and community-level factors

#### 3.9.1. Normalization of disrespect and abuse during childbirth

Moronkola et al [42] speculated that women who are not aware of their rights and have never been exposed to any other system of care are not sensitive to the disrespect and abuse of health care workers and see such behavior and attitude as status quo. These women are not likely to report being mistreated or disrespected.

#### 3.9.2. Financial barriers

An important barrier to respectful care at childbirth as well as skilled delivery service is the financial status of the woman [33–35, 39, 40, 42]. Some women indicated that they report to hospitals late or not at all because of the costs involved [33]. This might irk health providers who then react in an abusive and disrespectful way. One of the women in the qualitative study by Igboanugo et al [33] was quoted as having said “*Because most things that scare mothers away from such care …*, *is maybe the money involved*”. Another woman in the same study declared “*The nurses are harsh and rude when you don’t come to the hospital early enough…*..” In another study, women reported that they were also insulted when they could not pay their bills [34].

#### 3.9.3. Lack of autonomy and empowerment

Four studies found that majority of women reporting disrespect and abuse were uneducated and of low socioeconomic status [35, 37, 39, 42]. The authors of these studies concluded that educated women are better aware of their rights reducing the likelihood of being subjected to disrespectful and abusive behavior. Education may also increase self-confidence thereby reducing power differential between health providers and women [36]. Lamina et al [40] also commented on the higher rate of hospital deliveries in educated women postulating they were less likely to be disrespected thereby increasing their level of utilization.

### 3.10. National laws & policies, human rights, and ethics

#### 3.10.1. Lack of enforcement of national laws and policies and lack of legal redress mechanisms

None of the studies provided direct quantitative or qualitative evidence for this factor, however the discussion section of the study by Moore et al speculated that policies that protect the rights of women are hardly enforced and providers are rarely held accountable for their actions resulting in unchecked cases of disrespect and abusive [36].

### 3.11. Governance & leadership

#### 3.11.1. Lack of leadership/supervision for respect and non-abuse in childbirth

According to Igboanugo et al [33], sometimes, there is weak leadership and no supervision as there is nobody to check the providers’ actions in terms of the basic care that is delivered and the way patients are handled. One of the women in this study was quoted as follows: “*Sometimes it may be the consultant that is very rude*, *so there is nobody higher up to report to*” [33].

### 3.12. Providers & service delivery

#### 3.12.1. Provider distancing as a result of training

Provider training sometimes create separation between providers and patients by not giving much attention to the dynamics of interpersonal care[46]. This form of distancing can lead to provider insensitiveness as described by patients. Providers in a unique study were described as being used to illness and death leading to insensitiveness towards women during childbirth. *“Nothing touches their heart anymore”* was the way one of the women described providers’ insensitiveness to patients [34].

#### 3.12.2. Provider demoralization related to weak health systems

The negative effects of under-resourced and strained health systems on provider motivation were described as contributors to disrespect and abuse in three studies [33, 34, 37]. Bad attitude of health workers was attributed to health facilities being understaffed leading to them becoming overworked, tired and easily irritable [34]. Lack of training of staff to improve quality of care [33, 37], poor remuneration and lack of motivation [37] could contribute to negative attitude. Poorly designed hospital environment with minimal privacy and lacking equipment and infrastructure was also reported with as contributing factors to disrespect and abuse of women [33].

### 3.13. Consequences

The following consequences of disrespect and abuse were reported in the papers reviewed.

#### 3.13.1. Non utilization or delayed utilization of skilled delivery services

The bad attitude of health workers was cited as one major motivation for women avoiding skilled delivery services [34, 36, 39, 42], and choosing to deliver at home or in spiritual church based centers [35, 43]. Some women reported presenting to the hospital was a last resort if they had not attended antenatal or previously booked in the hospital because the health staff were usually very rude and abusive [33, 42].

#### 3.13.2. Psychological consequences

The lack of compassion and bad attitude of health workers was cited as resulting in social and psychological distance between the women and health workers reducing accessibility even when services were available [37].

## 4. Discussion

Our systematic review suggests that disrespect and abuse of women during childbirth occurs frequently in Nigeria, and can take the many forms described in the literature for other settings. The type of abuse most repeatedly reported was non-dignified care and the least commonly reported were physical abuse and detention in facilities, mentioned only in one study [8].This might be unsurprising as they are both extreme forms of abuse. Nevertheless, underreporting is possible as this behavior may be accepted as normal and not considered as abuse or disrespect by some women [29]. Thus such incidents may not have been spontaneously reported if specific questions about these situations were not asked by the researchers (27). Our findings should therefore not be interpreted as though such acts rarely exist.

Our review findings are consistent with the limited published research literature on disrespect and abuse of women during childbirth. Verbal and physical abuse, lack of privacy and confidentiality, maltreatment, detainment in facilities, and negative and unfriendly staff attitudes as a barrier to utilization of skilled delivery services have been reported [18, 21–23, 27, 29, 46, 47]. Similar to the findings of this review, factors such as inadequate staff, infrastructure, equipment and supplies and lack of supervision of providers have also been described as important factors that contribute to disrespect and abuse [12, 16, 18, 23].

The existence of disrespect and abuse was essentially defined as ‘unfriendly, poor or negative staff attitude’ in many studies [35, 36, 43, 45]. This vague description is open to interpretation and difficult to address without context specific descriptions on what exactly was considered unfriendly or negative. The qualitative and mixed method studies included in the review were more explicit and specific forms of disrespect and abuse experienced were described in more detail [33, 34, 41].

This is perhaps indicative that the nature of the topic under investigation requires an in-depth interaction with women who have had such experiences to fully explore their understanding of such issues. Qualitative and mixed method studies are more likely to achieve this purpose. The experiences of disrespect and abuse and the context in which it occurs differ and might not be easily captured on a questionnaire.

It is challenging to measure accurately the occurrence of disrespect and abuse. Combination of quantitative, qualitative and observational methods may be the best way as demonstrated by the USAID TRAction Project in Kenya (Heshima Project) [48] which combined questionnaire interviews of providers, in-depth interviews, focus group discussions and case narratives from women and observations of client provider interactions. The women interviewed reported less abuse than observed and as such, the review findings may grossly underestimate the occurrence of disrespect and abuse as it does not include studies with observations.

Only one study reported satisfactory and positive experiences from women [41], and more could be learned from these more positive encounters. Whilst past literature has shown that women can be both satisfied or happy with certain services and dissatisfied with other aspects of the care received, there was little nuance in the papers included in the review. For example, a woman may be grateful to have been saved but unhappy that providers discussed her case in public (26). This could be indicative of publication or researcher bias where negative experiences of women are mostly described or highlighted because these results are more interesting.

The Nigerian health system contributes to disrespect and abuse of women during childbirth by subjecting providers to degrading working conditions [33, 34, 37]. Women may sometimes perceive this system failure as an inadequacy on the provider’s part in term of long waiting times, lack of promptness of care, neglect or abandonment of care, lack of privacy, overcrowded rooms and sometimes provider’s lack of patience to indulge in pleasantries due to heavy work load. To achieve respectful and non-abusive care during childbirth, health systems must be responsive to specific needs of women at childbirth in a manner that ensures respect for their sexual and reproductive health and human rights[49].

Providers’ lack of training in interpersonal care is a critical factor influencing disrespect and abuse of women at childbirth [34, 46]. Studies have reported improvements in service provision and provider-client relations following training of healthcare providers on interpersonal and communication skills[50, 51]. This form of targeted training could improve providers’ self-awareness and sensitize them on how to better engage women during childbirth. In addition, counseling women in advance on what to expect during childbirth including their right to informed consent, privacy and confidentiality and inquiring if they have any preferences regarding labor and birth improves their birthing experience [51]

A respectful maternity care campaign (RMC) undertaken by White Ribbon Alliance Nigeria in 2013 resulted in a state level implementation plan in Kwara State to include RMC at every level of the state’s health system and approval of the RMC charter [52]. It also informed the development of the Nigeria-focused health workers training guide on RMC[53]. This is a good start to putting respectful maternity care on the policy radar in Nigeria. However, policies to promote RMC as a standard of practice are hardly adopted, rarely specific and do not translate into meaningful actions in numerous instances[49].

Moving forward, we believe disrespect and abuse of women at childbirth has not been extensively researched and comprehensively documented in Nigeria. The studies available do not explore the topic in details. As such, more research is needed to fully explore this issue. Use of standardized tools for quantitative measurements across all states, qualitative studies and observation of client provider interactions would prove beneficial.

### 4.1. Strengths and limitations

Due to the similarity with the review topic, using the Bowser and Hill landscape analysis on disrespect and abuse to inform the evidence synthesis was deemed appropriate and proved useful in structuring the results. However, there is a likelihood we missed out factors that the landscape analysis might not have covered. The analysis did not utilize systematic search approach and synthesis methodology and emphasized more behavioral issues and less structural issues such as providers working conditions and a narrative synthesis may have sufficed.

This review could also have benefited from a section reporting on how the authors of the studies reviewed defined the types of disrespect and abuse and how that fits into the Bowser and Hill framework. This would have helped to put the similarities and differences in perspective. However, such data was not available.

None of the studies involved husbands or other relatives who may be companions of these women during childbirth. Women, sometimes, may not be in the best position to describe or give account of their experiences as childbirth is a highly stressful period and companions may be more observant and their views very informative.

Most of the studies in this review did not indicate who administered the questionnaires and conducted the interviews. This is very important to the nature and context of the topic of this review. If providers were involved in the interviews, it is possible women were not be willing to divulge and voice their views and experiences leading to courtesy bias and underreporting of disrespect and abuse (26). There was also little information on providers’ perspective on disrespect and abuse in these studies. This is very important in understanding the causes and proffering solutions.

An important limitation is the medium to low quality of most of the studies inherent in the absence of power calculation, small sample size, risk of selection, recall and courtesy bias, absence of statistical significance and confidence intervals and their difficulty in accounting for potential confounders. The evidence provided is also not robust and appears superficial and lacking in context and depth. Because only a limited number of articles met our inclusion criteria, we chose to be inclusive regarding their methodological quality. For this reason, the findings of this review should be interpreted with caution.

## 5. Conclusion

This systematic review documented a broad range of disrespectful and abusive behavior experienced by women during childbirth in Nigeria, their contributing factors and consequences. Findings from this review contribute to the overall knowledge on barriers to the utilization of health facilities for delivery in Nigeria.

While the strength of the evidence is not robust, the nature of the factors influencing disrespectful and abusive behavior from the studies reviewed suggest that interventions such as empowering women and educating them on their rights, strengthening health systems to respond to specific needs of women at childbirth, improving providers training to include elements of interpersonal care and communication skills, and implementing and enforcing policies on respectful maternity care are important.

Further research is required to provide rigorous and more convincing evidence-base to explore disrespect and abuse of women during childbirth in Nigeria. This review explicitly points to the need for more robust evidence, particularly in the form of qualitative studies, since they are most valuable in terms of in-depth exploration of issues which this topic would benefit a lot from. Research should seek to understand the views and experiences of women, their families and providers. This will contribute immensely to the development and implementation of appropriate interventions.

## Supporting information

S1 Appendix Search strategies. Detailed search terms and filters applied to generate our search.
(DOCX)

S2 Appendix Quality appraisal for quantitative studies. (DOCX)

S3 Appendix Quality appraisal for qualitative studies. (DOCX)

S4 Appendix Study exclusion table. (DOCX)

S5 Appendix PRISMA checklist. (DOC)
