# Supplementary material for: Disrespect and abuse of women during childbirth in Nigeria: A systematic review
Source: PLoS One. 2017 Mar 21;12(3):e0174084. doi: 10.1371/journal.pone.0174084 (PMC5360318; doi:10.1371/journal.pone.0174084)
Supplement: S1 Appendix — Detailed search terms and filters applied to generate our search. (DOCX) [file pone.0174084.s001.docx]

# S1 Appendix. Search Strategies

**Medline** Searched 17/7/14

|  | **Key Word/MeSH Term  Search** | **Results** |
| --- | --- | --- |
| 1 | (((health provider* or nurse* or midwi* or doctor* or hospital staff*) adj3 (attitude* or behavio?r or practice*or relationship* or abuse or disrespect or neglect or discrimination or stigma or maltreatment or detention)) or quality of care or respectful care or non-consented care or non-confidential care or non-dignified care or humani#ation of birth).mp. | 51055 |
| 2 | Physician-Patient Relations/ | 61311 |
| 3 | exp Nurse-Patient Relations/ | 31181 |
| 4 | exp Patient Satisfaction/ | 62950 |
| 5 | exp "Attitude of Health Personnel"/ | 122575 |
| 6 | exp "Quality of Health Care"/ | 5064847 |
| 7 | exp Confidentiality/ | 44591 |
| 8 | exp Informed Consent/ | 36818 |
| 9 | exp Patient Care/ | 586997 |
| 10 | exp "Delivery of Health Care"/ | 809985 |
| 11 | 1 or 2 or 3 or 4 or 5 or 6 or 7 or 8 or 9 or 10 | 5718684 |
| 12 | ((birth* or intrapartum* or perinatal or childbirth* or labo?r or deliver* or obstetric* or matern* or pregnan*) adj3 (service* or facilit* or care* or hospital* or health centre* or health institution)).mp. | 147527 |
| 13 | exp Perinatal Care/ | 6749 |
| 14 | Delivery, Obstetric/ or Labor, Obstetric/ | 709834 |
| 15 | Pregnancy/ | 703308 |
| 16 | exp Parturition/ | 8459 |
| 17 | 12 or 13 or 14 or 15 or 16 | 828318 |
| 18 | nigeria* | 24846 |
| 19 | exp Nigeria/ | 21310 |
| 20 | (Nigeria or Anambra or Enugu or Akwa Ibom or Adamawa or Abia or Bauchi or Bayelsa or Benue or Bornu or Cross river or Delta or Ebonyi or Edo or Ekiti or Gombe or Imo or Jigawa or Kaduna or Kano or Katsina or Kebbi or Kogi or Kwara or Lagos or Nasarawa or Niger or Ogun or Ondo or Osun or Oyo or Plateau or Rivers or Sokoto or Taraba or Yobe or Zamfara or Abuja).mp. | 179204 |
| **21** | **18 or 19 or 20** | **180313** |
| **22** | **11 AND 17 AND 21** | **3186** |
| **23** | **limit 22 to yr="2004 -Current"** | **1258** |

**Embase** Searched 16/7/14

|  | **Key Word/MeSH Term  Search** | **Results** |
| --- | --- | --- |
| 1 | (((health provider* or nurse* or midwi* or doctor* or hospital staff*) adj3 (attitude* or behavio?r or practice*or relationship* or abuse or disrespect or neglect or discrimination or stigma or violence or maltreatment or detention)) or quality of care or respectful care or non-consented care or non-confidential care or non-dignified care or humani#ation of birth).mp. | 139008 |
| 2 | exp health personnel attitude/ | 132555 |
| 3 | exp midwife attitude/ | 179 |
| 4 | exp nurse attitude/ | 33221 |
| 5 | exp physician attitude/ | 41839 |
| 6 | exp physician assistant attitude/ | 29 |
| 7 | exp nurse patient relationship/ | 29948 |
| 8 | exp doctor patient relation/ | 80912 |
| 9 | exp health care quality/ | 1997645 |
| 10 | exp patient care/ | 536620 |
| 11 | exp confidentiality/ | 22351 |
| 12 | exp informed consent/ | 64746 |
| 13 | 1 or 2 or 3 or 4 or 5 or 6 or 7 or 8 or 9 or 10 or 11 or 12 | 2474353 |
| 14 | ((birth* or intrapartum* or perinatal or childbirth* or labo?r or deliver* or obstetric* or matern* or pregnan*) adj3 (service* or facilit* or care* or hospital* or health centre* or health institution)).mp. | 250874 |
| 15 | exp childbirth/ | 50957 |
| 16 | exp intrapartum care/ | 1076 |
| 17 | exp perinatal care/ | 40718 |
| 18 | exp labor/ | 36550 |
| 19 | exp birth/ | 20684 |
| 20 | exp delivery/ | 130074 |
| 21 | 14 or 15 or 16 or 17 or 18 or 19 or 20 | 419688 |
| 22 | nigeria* | 34251 |
| 23 | (Nigeria or Anambra or Enugu or Akwa Ibom or Adamawa or Abia or Bauchi or Bayelsa or Benue or Bornu or Cross river or Delta or Ebonyi or Edo or Ekiti or Gombe or Imo or Jigawa or Kaduna or Kano or Katsina or Kebbi or Kogi or Kwara or Lagos or Nasarawa or Niger or Ogun or Ondo or Osun or Oyo or Plateau or Rivers or Sokoto or Taraba or Yobe or Zamfara or Abuja).mp. | 163964 |
| 24 | exp Nigeria/ | 26684 |
| 25 | 22 or 23 or 24 | 166559 |
| **26** | **13 AND 21 AND 25** | **854** |
| **27** | **limit 26 to yr="2004 -Current"** | **636** |

**Cinahl** Searched 20/7/14

|  | **Key Word/MeSH Term  Search** | **Results** |
| --- | --- | --- |
| 1 | (((health provider* or nurse* or midwi* or doctor* or hospital staff*) N3 (attitude* or behavior or behaviour or practice* or relationship* or abuse or disrespect or neglect or discrimination or stigma or maltreatment or detention)) or quality of care or respectful care or non-consented care or non-confidential care or non-dignified care or humanization of birth) | 59082 |
| 2 | (MH "Physician Attitudes") | 9721 |
| 3 | MH "Attitude of Health Personnel+") | 57483 |
| 4 | (MH "Midwife Attitudes") | 1020 |
| 5 | MH "Nurse Attitudes") | 19222 |
| 6 | MH "Physician Assistant Attitudes") | 43 |
| 7 | MH "Quality of Health Care+") | 427780 |
| 8 | MH "Privacy and Confidentiality") | 15166 |
| 9 | MH "Physician-Patient Relations") | 20528 |
| 10 | MH "Nurse-Patient Relations") | 20584 |
| 11 | MH "Patient Abuse") | 1208 |
| 12 | 1 or 2 or 3 or 4 or 5 or 6 or 7 or 8 or 9 or 10 or 11 | 546351 |
| 13 | ((birth* or intrapartum* or perinatal or childbirth* or labor* or labour* or deliver* or obstetric* or matern* or pregnan*) N3 (service* or facilit* or care* or hospital* or health centre* or health institution)) | 81758 |
| 14 | MH "Labor+") | 8030 |
| 15 | MH "Intrapartum Care+") | 4912 |
| 16 | MH "Perinatal Care") | 2183 |
| 17 | MH "Childbirth+") | 16558 |
| 18 | MH "Delivery, Obstetric+") | 7544 |
| 19 | MH "Pregnancy+") | 123397 |
| 20 | MH "Maternal Health Services+") | 17154 |
| 21 | 13 or 14 or 15 or 16 or 17 or 18 or 19 or 20 | 192720 |
| 22 | (nigeria*) | 4613 |
| 23 | (Nigeria or Anambra or Enugu or Akwa Ibom or Adamawa or Abia or Bauchi or Bayelsa or Benue or Bornu or Cross river or Delta or Ebonyi or Edo or Ekiti or Gombe or Imo or Jigawa or Kaduna or Kano or Katsina or Kebbi or Kogi or Kwara or Lagos or Nasarawa or Niger or Ogun or Ondo or Osun or Oyo or Plateau or Rivers or Sokoto or Taraba or Yobe or Zamfara or Abuja) | 9870 |
| 24 | MH "Nigeria") | 4229 |
| 25 | 22 or 23 or 24 | 9870 |
| **26** | **12 AND 21 AND 25** | **130** |
| **27** | **LIMIT TO JAN 2004** | **118** |

**Africa Wide Information** Searched 20/7/14

|  | **Key Word Search** | **Results** |
| --- | --- | --- |
| 1 | (((health provider* or nurse* or midwi* or doctor* or hospital staff*) N3 (attitude* or behavior or behaviour or practice* or relationship* or abuse or disrespect or neglect or discrimination or stigma or maltreatment or detention)) or quality of care or respectful care or non-consented care or non-confidential care or non-dignified care or humanization of birth) | 4541 |
| 2 | ((birth* or intrapartum* or perinatal or childbirth* or labor* or labour* or deliver* or obstetric* or matern* or pregnan*) N3 (service* or facilit* or care* or hospital* or health centre* or health institution)) | 34734 |
| 3 | **(**Nigeria or Anambra or Enugu or Akwa Ibom or Adamawa or Abia or Bauchi or Bayelsa or Benue or Bornu or Cross river or Delta or Ebonyi or Edo or Ekiti or Gombe or Imo or Jigawa or Kaduna or Kano or Katsina or Kebbi or Kogi or Kwara or Lagos or Nasarawa or Niger or Ogun or Ondo or Osun or Oyo or Plateau or Rivers or Sokoto or Taraba or Yobe or Zamfara or Abuja) | 148964 |
| **4** | **1 AND 2 AND 3** | **98** |
| **5** | **LIMIT TO JAN 2004** | **75** |

**Popline** Searched 18/7/14

|  | **Key Word Search** | **Results** |
| --- | --- | --- |
| 1 | (health provider* OR nurse* OR midwi* OR doctor* OR hospital staff* OR attitude* OR behavior OR behaviour OR practice* OR relationship* OR abuse OR disrespect OR neglect OR discrimination OR stigma OR maltreatment OR detention OR quality of care OR respectful care OR non-consented care OR non-confidential care OR non-dignified care OR humanization of birth) |  |
| 2 | (birth* OR intrapartum* OR perinatal OR childbirth* OR labor OR labour OR deliver* OR obstetric* OR matern* OR pregnan* OR service* OR facilit* OR care* OR hospital* OR health centre* OR health institution) |  |
| **4** | **1 AND 2** | **28** |
