# Supplementary material for: Disrespect and abuse of women during childbirth in Nigeria: A systematic review
Source: PLoS One. 2017 Mar 21;12(3):e0174084. doi: 10.1371/journal.pone.0174084 (PMC5360318; doi:10.1371/journal.pone.0174084)
Supplement: S2 Appendix — (DOCX) [file pone.0174084.s002.docx]

## **S2 Appendix. Quality Appraisal Table for Quantitative Studies**

|  | **Uzochukwu et al, 2004** | **Udoma E.J et al, 2008** | **Moronkola O A et al, 2007** | **Moore BM et al, 2011** | **Iyaniwura et al, 2009** | **Lamina MA et al, 2004** | **Onah HE et al, 2006** | **Sule et al, 2012** | **Chigbu et al, 2011** | **Idris et al, 2013** | **Okafor et al 2015** | **Nnebue et al 2014** | **Ashimi et al 2015** |
| --- | --- | --- | --- | --- | --- | --- | --- | --- | --- | --- | --- | --- | --- |
| **1. Did the study address a clearly focused question / issue?** | Yes, it was stated | Yes | Yes | Yes | Yes | Yes | Yes | Yes | Yes | Yes | Yes | Yes | Yes |
| **2. Is the research method (study design) appropriate for answering the research question?** | Yes, authors utilized a mixed method approach. | Yes | Yes | Yes | Yes | Yes | Yes | Yes | Yes | Yes | Yes | Yes | Yes |
| **3. Is the method of selection of the subjects clearly described?** | Yes, random sampling and purposive sampling used | No, not clearly detailed | No, not clearly detailed | Yes | Yes | No, not clearly detailed | Yes, multistage sampling | No | No | Yes | Yes | No | Yes |
| **4. Could the way the sample was obtained introduce (selection) bias?** | Maybe, houses with no women who had delivered in the last 12 months were excluded. | Yes, criteria for selection of the 47 spiritual clinics used not clear | Yes, criteria for selection of the 11 local governments used not clear | No | No | Yes | No | Yes | Yes | No | Yes | Yes | No |
| **5. Was the sample of subjects representative with regard to the population to which the findings will be referred?** | Yes | No, not clearly detailed | No, not clearly detailed | Yes | Yes | No, not clearly detailed | Yes | No | No, facility based study | yes | No | No | Yes |
| **6. Was the sample size based on pre-study considerations of statistical power?** | Sample size given but no information on power calculation | Sample size given but no information on power calculation | Sample size given but no information on power calculation | Sample size given but no information on power calculation | Yes, sample size and power calculation given | No, sample size not given. | Yes | No | No | Yes | Yes | Yes | Yes |
| **7. Was a satisfactory response rate achieved?** | Not certain, final number of respondents not clearly stated | Yes | Yes | Yes | Yes | Not certain | Yes | Yes | Yes | Yes | Yes | Yes | Yes |
| **8. Are the measurements (questionnaires) likely to be valid and reliable?** | Yes, questionnaires were pre tested | No, not pre-tested | Yes, pretested | No, not clearly detailed | Yes, questionnaires pretested and interviewers trained | No | Yes, pretested, pilot study and interviewers trained | Yes | Yes | Yes | Yes | No | Yes |
| **9. Was the statistical significance assessed?** | Yes | No | Yes | No | Yes | No | Yes | Not for all variables | Yes | Yes | Yes | Yes | Yes |
| **10. Are confidence intervals given for the main results?** | No | No | No | No | No | No | No | No | No | Yes | Yes | No | Yes |
| **11. Could there be confounding factors that haven’t been accounted for?** | Yes, some variables were accounted for but there could still be some unknown confounders. | Yes | Most of the variables accounted for | Most of the variables accounted for | Yes | Yes | Most of the confounders accounted for, however unknown confounders may still exist. | Yes | Yes | Yes | Yes | Yes | Yes |
