# Supplementary material for: Disrespect and abuse of women during childbirth in Nigeria: A systematic review
Source: PLoS One. 2017 Mar 21;12(3):e0174084. doi: 10.1371/journal.pone.0174084 (PMC5360318; doi:10.1371/journal.pone.0174084)
Supplement: S3 Appendix — (DOCX) [file pone.0174084.s003.docx]

## **S3 Appendix. Quality Appraisal Table for Qualitative Studies**

| **Appraisal questions** | **Igboanugo et al, 2011** | **Uzochukwu et al, 2004** | **Nnebue et al** |
| --- | --- | --- | --- |
| **1. Was there a clear statement of the aims of the research?** | Yes, it was clearly stated | Yes | Yes |
| **2. Is a qualitative methodology appropriate?** | Yes, the study was interested in the perception of maternity services by the women | Yes, it provided more in depth understanding of the issues explored with the questionnaire. | Yes |
| **3. Was the research design appropriate to address the aims of the research?** | Yes, the design was qualitative in tandem with the aims | Yes | Yes |
| **4. Was the recruitment strategy appropriate to the aims of the research?** | Yes, purposive sampling of informants. | Yes, purposive sampling of informants | Yes, purposive |
| **5. Was the data collected in a way that addressed the research issue?** | Yes, semi structured interviews with interviewer prompts | Yes | Yes |
| **6. Has the relationship between researcher and participants been adequately considered?** | Yes | Yes | No |
| **7. Have ethical issues been taken into consideration?** | Yes, informed written consent and informants afforded anonymity and confidentiality | Ethical approval received but no information on informed consent or confidentiality | No |
| **8. Was the data analysis sufficiently rigorous?** | Yes, transcribed and coded. | Yes, content analysis employed | No |
| **9. Is there a clear statement of findings?** | Yes | Yes | Yes |
| **10. How valuable is the research?** | High | High | Yes |
