# Supplementary material for: Disrespect and abuse of women during childbirth in Nigeria: A systematic review
Source: PLoS One. 2017 Mar 21;12(3):e0174084. doi: 10.1371/journal.pone.0174084 (PMC5360318; doi:10.1371/journal.pone.0174084)
Supplement: S4 Appendix — (DOCX) [file pone.0174084.s004.docx]

**S4 Appendix. Study Exclusion Table**

| **Study** | **Study Focus** | **Reason For Exclusion** |
| --- | --- | --- |
| Adekunle OO, Similoluwa AO. Determinants of place of delivery among booked patients in a tertiary institution. Annals of African Medicine. 2012;11(3):192-3. | To determine what guides the choice of the place of delivery of the booked patients in A.B.U. Teaching Hospital, Zaria. | Did not document quality of care issues relating to any form of disrespect or abuse of women at childbirth |
| Oyo-Ita AE, Etuk SJ, Ikpeme BM, Ameh SS, Nsan EN. Patients' perception of obstetric practice in Calabar, Nigeria. Nigerian Journal of Clinical Practice. 2007; 10(3):224-8. | To determine the influence of patients perception of obstetric practice on low utilization of health facilities for delivery. | The study focused more on opinions of mothers towards certain birth practices and what their preferences were. There was minimal report of actual experiences of women with providers at childbirth. |
| Akin-Otiko BO, Bhengu BR. Client education experiences and expectations of women at the first level of maternal and child care in Kaduna state, Nigeria. Midwifery. 2012;28(6):e893-9. | To explore the client education experiences of women at first level maternal and child health-care facilities in Kaduna State Nigeria. | The study explored experiences as related to client education practices of maternity centres in the region and there was no mention of any form of disrespect and abuse. |
| Akpabio, II, Edet OB, Etifit RE, Robinson-Bassey GC. Women's preference for traditional birth attendants and modern health care practitioners in Akpabuyo community of Cross River State, Nigeria. Health Care for Women International. 2014;35(1):100-9. | To determine proportion of women who preferred traditional birth attendants (TBA) and their reason. | The study did not include any relevant data on any form of disrespect and abuse by skilled birth attendants, the majority of the results focused on experiences with traditional birth attendants. |
| Chigbu CO, Ezenyeaku CC. Women's opinions and experiences with induction of labor and cesarean delivery on request in south eastern Nigeria. International Journal of Gynaecology & Obstetrics. 2008;103(2):158-61. | To assess the opinions and experiences of women regarding induction of labour and Caesarean delivery on request in south eastern Nigeria. | There was little emphasis on experiences of disrespect and abuse and more on opinions. |
| Bawa SB, Umar US, Onadeko M. Utilization of obstetric care services in a rural community in southwestern Nigeria. Afr J Med Med Sci. 2004;33(3):239-44. | To study the pattern of utilization of antenatal, delivery and postnatal care services in the community. | Did not document quality of care issues relating to any form of disrespect or abuse of women at childbirth |
| Opara JA, Ellah AO. Women's Perception Of Maternal And Child Health Care Services [MCH]. Journal of Technology and Education in Nigeria. 2007;12 (2):pp. 19-24. | To understand the perception of women on maternal and child health care services obtained from Federal Medical Center Owerri | The study focused on quality of education topics and family planning methods with minimal emphasis on care received at childbirth. |
| Abiodun AJ. Patients' satisfaction with quality attributes of primary health care services in Nigeria. Journal of Health Management. 2010;12(1):39-54. | To determine the quality attributes that determine overall satisfaction with care. | The study was not particular to women at childbirth |
| Doctor HV, Dahiru T. Utilization of non-skilled birth attendants in northern nigeria: a rough terrain to the health-related mdgs... Millennium and Development Goals. African Journal of Reproductive Health. 2010;14(2):37-45. | To study the reasons for low utilisation of antenatal and delivery care among women with recent pregnancies, and the socio-cultural beliefs and practices that influenced them | Did not document quality of care issues relating to any form of disrespect or abuse of women at childbirth |
| Ezeonwu MC. Maternal Birth Outcomes: Processes and Challenges in Anambra State, Nigeria. Health Care for Women International. 2011;32(6):492-514. | To explore the views of nurse experts and policymakers on maternal birth outcomes in Anambra State. | Did not document quality of care issues relating to any form of disrespect or abuse of women at childbirth |
| Fawole AO, Okunlola MA, Adekunle AO. Clients' perceptions of the quality of antenatal care. Journal of the National Medical Association. 2008;100(9):1052-8. | To assess perceptions of pregnant women about quality of antenatal care. | Only explored views regarding antenatal care received. |
| Oladapo OT, Iyaniwura CA, Sule-Odu AO. Quality of antenatal services at the primary care level in southwest Nigeria. African Journal of Reproductive Health. 2008;12(3):71-92. | To assess their perspectives on the quality of antenatal care received. | Only explored views regarding antenatal care received. |
| Asuquo EEJ, Etuk SJ, Duke F. Staff Attitude as a Barrier to the Utilisation of University of Calabar Teaching Hospital for Obstetric Care. African Journal of Reproductive Health. 2000;4(2):69-73. | To assess how the attitudes of hospital staff influence the utilisation of health facility for obstetric care at the University of Calabar Teaching Hospital (UCTH), Calabar, Nigeria | Studies earlier than 2004 were excluded. |
| Adegoke AA, Mani S, Abubakar A, van den Broek N. Capacity building of skilled birth attendants: a review of pre-service education curricula. Midwifery. 2013;29(7):e64-72. | To assess the level, type and content of pre-service education curricula of health workers providing maternity service. | Study focused on education and training of providers. Did not document quality of care issues relating to any form of disrespect or abuse of women at childbirth |
| Adekanye AO, Adefemi SA, Okuku AG, Onawola KA, Adeleke IT, James JA. Patients' satisfaction with the healthcare services at a north central Nigerian tertiary hospital. Niger J Med. 2013;22(3):218-24. | To determine the level of satisfaction of patients with the healthcare services at Federal Medical Centre, Bida (FMCB) Nigeria and the factors associated with patients' satisfaction. | Studies on general patient population without distinguishing women at childbirth. |
| Aniebue UU, Aniebue PN. Women's perception as a barrier to focused antenatal care in Nigeria: the issue of fewer antenatal visits. Health Policy & Planning. 2011;26(5):423-8. | To examine the attitude of pregnant women to a new antenatal care model in Enugu, Nigeria. | Only explored views regarding antenatal care received. |
| Babalola S, Fatusi A. Determinants of use of maternal health services in Nigeria--looking beyond individual and household factors. BMC Pregnancy & Childbirth. 2009;9:43. | To examine the determinants of maternal services utilization in Nigeria, with a focus on individual, household, community and state-level factors | Only explored views regarding antenatal care received. |
| Enabudoso E, Isara AR. Determinants of patient satisfaction after cesarean delivery at a university teaching hospital in Nigeria. International Journal of Gynaecology & Obstetrics. 2011;114(3):251-4. | To assess the prevalence of satisfaction, and associated factors, among parturients who had recently delivered by cesarean. | Explored views of women on caesarian section and not on providers attitude during the procedure |
| Ezechi OC, Fasubaa OB, Obiesie LO, Kalu BK, Loto OM, Dubub VI, et al. Delivery outside hospital after antenatal care: prevalence and its predictors. J Obstet Gynaecol. 2004;24(7):745-9. | To assess why women booked for antenatal care at the two tertiary hospitals of the Obafemi Awolowo University Teaching Hospital Complex, Ile Ife,-Nigeria defaulted from hospital delivery | No mention of disrespect and abuse of women at childbirth. |
| Ike SO. Work attitudes and perceptions of South Eastern Nigerian health workers. West African Journal of Medicine. 2008;27(3):164-6. | To evaluate the work attitudes and perceptions of health workers in South Eastern Nigeria in one of the biggest Federal Teaching Hospitals in Nigeria. | Emphasis on training and workers and no mention of provider client interactions |
| Nsemo AD, John ME, Etifit RE, Mgbekem MA, Oyira EJ. Clinical nurses' perception of continuing professional education as a tool for quality service delivery in public hospitals Calabar, Cross River State, Nigeria. Nurse Education in Practice. 2013;13(4):328-34. | To determine the perception of nurses on various aspects of continuing professional education (CPE) | Focus on educational training of nurses. |
| Olusanya BO, Alakija OP, Inem VA. Non-uptake of facility-based maternity services in an inner-city community in Lagos, Nigeria: an observational study. Journal of Biosocial Science. 2010;42(3):341-58. | To establish the pattern and uptake of maternity services and associated factors against the backdrop of rapid urbanization in Nigeria | Only explored views regarding antenatal care received. |
